# The effect of post-activation potentiation on neuromuscular activation of smashing technique during the recovery period of meniscal injuries in elite badminton players: non-negative matrix factorization-based muscle and time-frequency coherence

**DOI:** 10.3389/fphys.2026.1752266

**Published:** 2026-03-05

**Authors:** Hongkai Zhuang, Siyao Hong, Yi Xia, Yi Sheng

**Affiliations:** School of Athletic Performance, Shanghai University of Sport, Shanghai, China

**Keywords:** after-effect, high-level badminton athletes, meniscal injury, muscle synergy, neuromuscular electrical stimulation

## Abstract

**Objectives:**

To investigate the effects of three distinct post-activation potentiation (PAP) interventions—neuromuscular electrical stimulation (NMES), elastic band resistance, and squats—on neuromuscular activation during the smashing technique in high-level badminton athletes recovering from meniscus injuries. Furthermore, to elucidate the underlying mechanisms at the neuromotor control level through analyses of muscle synergy and intermuscular coherence.

**Methods:**

Eighteen high-level male badminton athletes in the recovery phase of meniscus injuries were recruited. Surface electromyographic signals were recorded during forehand smash execution following respective interventions: squats, elastic band resistance, and NMES. Non-negative matrix factorization (NMF) analyzed muscle synergies, extracting synergistic module counts, muscle weights, and activation duration parameters. Time-frequency coherence (TFC) was calculated for specific muscle pairs.

**Results:**

The resistance band group (RBG) exhibited a significantly higher number of synergies (5.0 ± 0.63) compared to the squat group (SG) (3.33 ± 0.52, p = 0.005) and the electrical stimulation group (ESG) (2.33 ± 0.82, p < 0.001). In terms of muscle activation weights, the ESG showed markedly increased contributions from key lower limb muscles across multiple synergy modules. E.g., in SYN4, activation weights for gastrocnemius medialis (GM) and lateralis (GL) in the ESG (GM: 0.25 ± 0.31; GL: 0.28 ± 0.28) were significantly higher than in the SG (GM: 0.08 ± 0.20; GL: 0.06 ± 0.09) (p < 0.05), representing an increase exceeding 200%. Intermuscular coherence analysis revealed that the ESG demonstrated superior coherence across α, β, and γ bands for several trunk–limb muscle pairs. E.g., within the α band, the biceps BB–LD pair in the ESG was significantly higher than in both the SG (p = 0.002) and the EBG (p = 0.007).

**Conclusion:**

Neuromuscular electrical stimulation effectively optimizes muscle coordination patterns during smash execution in athletes recovering from meniscal injuries. It enhances activation of key muscle groups and multi-band neural coordination, representing an efficient rehabilitation strategy for neuromuscular control function optimization.

## 1 Introduction

In competitive sports, the prevention and rehabilitation of sports injuries are central to safeguarding athletes’ careers and optimizing performance. Badminton, as a high-intensity, multi-directional net-based sport, involves technical demands such as frequent abrupt stops, rapid starts, directional changes, and lunging movements. These place exceptional stress on the stability and load-bearing capacity of the knee joint, making knee injuries one of the most prevalent types in this discipline (Yu et al., 2023). Among these, meniscal injuries are particularly prevalent (Yu et al., 2023; Shariff et al., 2009). As a vital cushioning structure within the knee joint, meniscal damage can directly cause joint pain, locking, and reduced stability (Coombes et al., 2024), severely limiting an athlete’s competitive performance. The rehabilitation process following a meniscal injury, especially the later functional recovery phase, is crucial for enabling an athlete’s return to competition (VAN MELICK). Traditional rehabilitation approaches, after restoring basic function, often struggle to effectively activate the neuromuscular system and enhance explosive power. Consequently, athletes returning to competition frequently face persistent limitations in movement and force generation (Culvenor et al., 2015). Therefore, exploring more efficient rehabilitation training methods holds significant importance for shortening the recovery period following meniscal injuries and enhancing athletes’ sporting performance.

**TABLE 1:** Subject characteristics (n = 18).

Age (years)	Height (cm)	Weight (kg)	Years of training (years)
22.8 ± 3.2	178.5 ± 5.1	72.3 ± 4.7	10.5 ± 2.8

In recent years, post-activation potentiation (PAP) has emerged as a research focus within the field of athletic training. This physiological phenomenon involves subjecting the neuromuscular system to brief, high-intensity loading stimuli, thereby transiently enhancing muscle strength and explosive power (Hodgson et al., 2005). Its theoretical core lies in utilizing short-duration, high-intensity muscle contractions to elevate neuromuscular excitability, muscle fiber recruitment efficiency, and explosive power, ultimately translating into improved athletic performance (Hodgson et al., 2005). Existing research confirms that employing high-load strength training, combined with 4–16 min of rest intervals, effectively alleviates fatigue and stimulates PAP, thereby enhancing athletic performance (Arabatzi et al., 2014). Among these, the squat stands as one of the most prevalent methods within Constant Resistance Training (CRT), also termed free weight training. By employing high-load, low-repetition stimuli, it maximally activates the nervous system and fast-twitch muscle fibers, representing a classic and highly effective approach for inducing the post-activation potentiation effect (Wilson and Flanagan, 2008). Variable Resistance Training (VRT), combining elastic bands with free weights, dynamically adjusts external load to stimulate neural adaptation (Frost et al., 2010), thereby optimizing muscular strength performance. It is noteworthy that while the lateral band exercise employed in this study is a frontal-plane, low-load movement, evidence suggests such training can enhance hip stabilizer activation and rate of force development, potentially benefiting sagittal-plane explosive actions like the badminton smash through improved neuromuscular control and force transmission (Medeiros et al., 2022). This exercise highly adapts to the ascending characteristics of strength curves; Electrical stimulation combined with squat training refers to a composite training method where, whilst performing weighted squats, specific frequency and intensity electrical pulses are applied to major lower-limb muscle groups via external electrical stimulation. This enhances muscle activation and improves training outcomes (Lin et al., 2025).

In badminton, the smash technique serves not only as a crucial means of scoring during matches but also as an ideal model for assessing an athlete’s overall neuromuscular coordination and control capabilities. This complex movement sequence originates from the lower limbs’ push-off and jump, progresses through core muscle stabilization and force transmission, and culminates in the upper limbs’ whip-like power generation. It thus fully demonstrates the efficient transfer of the kinetic chain and the precise coordination of multiple muscle groups (Matsunaga and Kaneoka, 2018). Consequently, the quality of the smash directly reflects an athlete’s explosive power, stability, and neuromuscular control, serving as a primary indicator for evaluating their athletic performance.

The rationale for employing muscle synergy analysis via NMF in this cohort is underpinned by the neuroadaptive changes that accompany lower limb joint pathology. Following injuries such as meniscal tears, pain, effusion, and mechanical instability disrupt proprioceptive feedback and alter central motor commands. This often precipitates a fundamental reorganization of locomotor muscle synergies—the modular building blocks of movement—as the nervous system seeks compensatory strategies to maintain function. Recent evidence highlights such adaptations in similar conditions. For instance, a study on chronic ankle instability demonstrated altered synergy structure and coordination during landing, illustrating how the neuromuscular system recalibrates module composition in response to joint impairment (Jie et al., 2024). Complementing this, research on anterior cruciate ligament (ACL) deficient knees has also identified significant modifications in muscle synergy complexity during gait, further underscoring that ligamentous knee injuries directly impact motor control organization (Sheikhi et al., 2021). These findings provide a compelling parallel, suggesting that meniscal injury likely induces comparable alterations in synergy recruitment during complex, sport-specific tasks like the badminton smash. Therefore, applying NMF offers a powerful framework to objectively quantify these neuroadaptive changes and evaluate the efficacy of different rehabilitation interventions.

Building upon this understanding of pathological synergy reorganization, This study, set within the rehabilitation phase following meniscal injuries in elite badminton athletes, aims to investigate the effects of different post-activation potentiation (PAP) intervention protocols on the neuromuscular activation characteristics during the smash technique. Building upon muscle synergy theory, the study will employ a combined approach of muscle synergy analysis and intermuscular coherence analysis. This will enable a quantitative comparison of activation patterns, synergy strategies, and neuromuscular coordination within key lower-limb and trunk muscle groups during the smash movement across different PAP interventions. Consequently, it will elucidate the underlying mechanisms for enhancing sport-specific performance at the neuromotor control level, thereby providing theoretical and experimental foundations for optimizing rehabilitation protocols for elite athletes.

## 2 Participants and methods

### 2.1 Participants

This study included 18 high-level male badminton athletes in the recovery phase of unilateral knee meniscus injury. All participants held national athlete status and had competed in high-level tournaments including national championships, with an average professional training duration of 10.5 ± 2.8 years (Table 1). Their mean age was 22.8 ± 3.2 years, mean height 178.5 ± 5.1 cm, and mean weight 72.3 ± 4.7 kg. To ensure homogeneity of subjects and safety of the intervention study, inclusion criteria were as follows:

1. Injury screening criteria. All subjects were clinically diagnosed via MRI within 6 months prior to enrolment as having unilateral Grade II meniscal tears in the knee, presenting as partial meniscal tears accompanied by persistent pain and functional joint instability. There were no clear indications for surgery, rendering them suitable for conservative rehabilitation treatment (Vrgoč et al., 2023).
2. Rehabilitation phase definition criteria. All subjects were in the mid-to-late stages of systematic rehabilitation, having completed foundational functional recovery training. They possessed basic weight-bearing and movement capabilities, with no acute inflammatory manifestations such as joint redness, swelling, or effusion, and a visual analogue scale (VAS) pain score ≤3 points.
3. Exclusion criteria. To ensure homogeneity of the sample and isolate the effects of PAP on conservatively managed meniscal injuries, subjects with any history of knee surgery (including but not limited to meniscectomy, meniscal repair, ligament reconstruction) were excluded. Additional exclusions included concomitant major knee joint structural injuries (e.g., anterior cruciate ligament, collateral ligaments), neurological disorders, or bilateral knee pathology (van Melick et al., 2016).

Although athletes demonstrate recovery of basic functional abilities during the late phase of meniscal injury rehabilitation, their neuromuscular control, explosive power, and multi-muscle coordination are often not fully rebuilt. This significantly impairs their specialized technical performance and competitive readiness upon returning to competition. Therefore, introducing scientifically grounded Post-Activation Potentiation (PAP) intervention at this stage, which promotes functional remodeling of movement patterns through neuromuscular activation, holds clear clinical and practical training significance. This study received ethical approval from Shanghai University of Sport (Approval No.: **102772025RT064**), and all participants signed informed consent forms.

### 2.2 Experimental equipment

**FIGURE 1:**
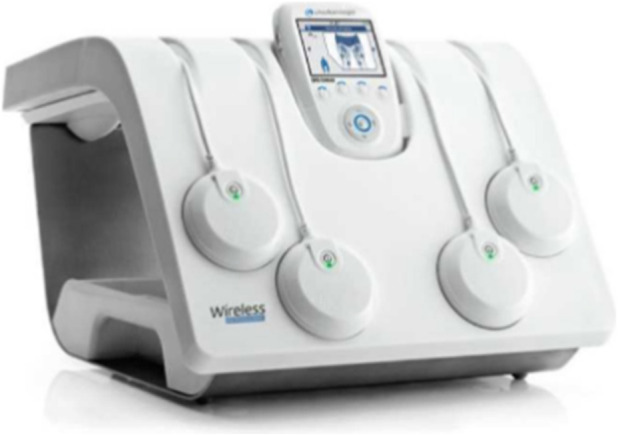
Compex SP 8.0 electrical stimulation device.

**FIGURE 2:**
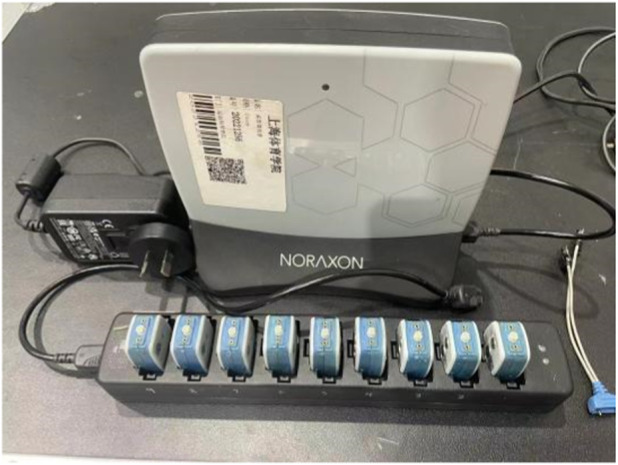
Noraxon wireless surface electromyography.

**FIGURE 3:**
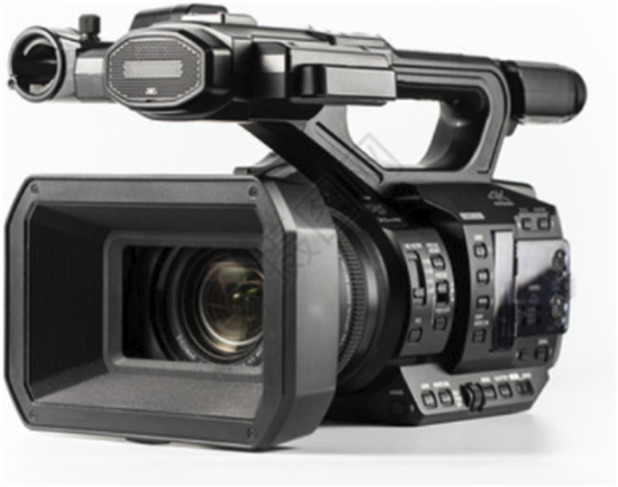
High-speed camera.

1. Compex SP 8.0 electrical stimulation device. NMES stimulation employed the Compex SP 8.0 electrical stimulation device, utilizing a dual-symmetrical square wave with a frequency range of 1–120 Hz and pulse widths of 200–400 μs (Figure 1).
2. Noraxon Wireless Surface Electromyography. This study employed the Ultium EMG wireless surface electromyography system manufactured by Noraxon Inc. of the United States for signal acquisition. Operating at a sampling frequency of 2000 Hz, the system’s integrated bipolar Ag/AgCl surface electrodes and miniature wireless transmitters effectively eliminate cable interference, rendering it particularly suitable for the precise capture of specialized technical movements such as those performed in badminton (Figure 2).
3. High-speed camera. A 200 Hz model was employed, undergoing three-dimensional spatial calibration prior to experimentation using a 12-point calibration frame (1 × 1 × 0.8 m), with reprojection error <0.3 mm (Figure 3).
4. Intervention Equipment. (a) Barbell. The Swedish Eleiko professional training barbell and matching weight plates were selected for performing squats, providing stability and ensuring experimental safety. (b) Resistance Bands. Umay resistance bands were chosen to combine variable resistance with squat movements, altering load curves and force patterns for resistance band-assisted squats. (c) Badminton rackets. Standardized equipment control protocols were applied: all subjects performed smash tests using uniformly provided and calibrated rackets (YONEX/NANOFLARE 800 GAME) supplied by researchers to ensure consistent performance and comparability. (d) Badminton shuttlecock. YONEX brand shuttlecocks, model AS05, were employed. This batch was procured uniformly to meet experimental requirements, ensuring their weight, flight velocity, and aerodynamic stability complied with testing standards.

### 2.3 Testing procedures

1. Maximum Voluntary Contraction (MVC) Test: Prior to formal testing, each target muscle undergoes three MVC tests. The maximum value is recorded for electromyographic signal normalisation.
2. Badminton Forehand Smash Test: Subjects shall execute five forehand smashes at match speed on a standard badminton court (balls delivered by a ball machine), targeting a 2 m × 1.5 m rectangular area on the opponent’s court. Electromyographic and kinematic data shall be collected synchronously.

### 2.4 Intervention methods and intensity

A randomized crossover design was employed, with all participants receiving three intervention treatments at 7-day intervals. A standardized warm-up preceded each intervention session (Table 2).

1. Squat Group (SG): Feet positioned 1.2–1.5 times shoulder-width apart, toes abducted 10°–15°, barbell placed on upper trapezius. Squat depth controlled at 90°–100° knee angle relative to the ground. Load set at 70% of 1-rep maximum (1RM), performed in three sets of three repetitions with 2-min rest intervals. Testing conducted 8 min post-intervention.
2. Resistance Band Group (RBG): A gold-grade TheraBand® Professional Resistance Band Loop (Performance Health, Akron, OH, United States of America) was secured above participants’ knee joint while performing side steps. The intervention load was standardized by elongating the band to 200% of its resting length in a standardized starting position (feet shoulder-width apart, knees flexed at approximately 30°). According to the manufacturer’s force-elongation specifications, this setup provides a nominal resistance of approximately 13.0 kg-force (LeBoff et al., 2022). Pilot testing confirmed that this resistance equated to an average external load of 18% of the participants’ body weight (Zhang et al., 2025). Three sets of 10 m were completed with 2 min’ rest between sets. Testing occurred 6 min post-intervention.
3. Electrical Stimulation Group (ESG): Electrical stimulation (75Hz, 400 μs, 90% tolerable intensity) was applied when the knee joint reached 90° flexion during squats, continuing until movement completion. Load intensity was set at 70% of 1RM. Perform three sets of three repetitions, with 2 min rest between sets. Testing was conducted 6 min post-intervention.

### 2.5 Data acquisition

A Noraxon wireless surface electromyography system (sampling frequency 2000 Hz) was synchronized with a 200 Hz high-speed camera to concurrently capture electromyographic and kinematic data during the smash action. Target muscles included: deltoid (DEL), biceps brachii (BB), triceps brachii (TB), brachioradialis (BRD), Gastrocnemius medial head (GM), Gastrocnemius lateral head (GL), Vastus medialis (VM), Vastus lateralis (VL), Biceps femoris (BF), Gluteus maximus (GLM), Rectus abdominis (ABS), Latissimus dorsi (LD), Trapezius (TRAP), and Pectoralis major (PM). Prior to testing, skin was cleansed with 75% alcohol. Electrodes were applied following SENIAM guidelines and secured with medical adhesive tape.

**FIGURE 4:**
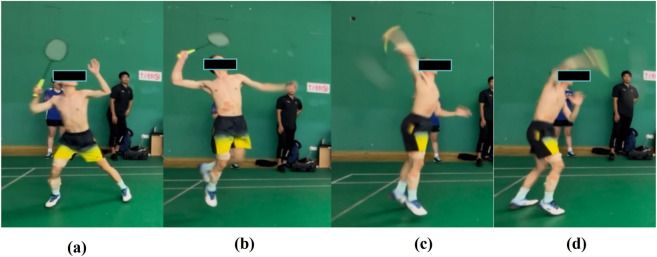
Phases of the badminton smash stroke **(a)** is Movement phase; **(b)** is take-off phase; **(c)** is Backswing phase; **(d)** is Ball strike phase.

Based on the biomechanical characteristics of the badminton smash action, a complete smash motion is divided into four consecutive phases (Figure 4):

1. Movement phase: From the initiation of movement (a) to the take-off moment (b).
2. Take-off phase: From the take-off moment (b) to the backswing initiation (c).
3. Backswing phase: From the backswing initiation (c) to the ball contact moment (d).
4. Ball strike phase: From ball strike moment (d) to follow-through completion moment (e).

Electromyographic signals from designated muscles were recorded during the smash. Muscle coordination characteristics were subsequently extracted via coherence analysis and non-negative matrix factorisation (NMF), investigating the effects of different interventions on muscle coherence and coordination patterns.

### 2.6 Data processing

Muscle synergies and intermuscular coherence analysis were performed using R software (version 4.2.0), the muscle synergies v1.2.5 package, and custom Python scripts. The specific workflow is as follows.

#### 2.6.1 Data extraction and preprocessing

Data synchronously acquired from a 200 Hz high-speed camera and a surface electromyography system with a 2000 Hz sampling frequency were processed. Each complete smash cycle (from take-off to follow-through completion) was defined based on kinematic features. Corresponding high-frequency electromyography data segments were extracted from the synchronized time signals for subsequent analysis. A fourth-order Butterworth bandpass filter (20–400 Hz) was applied to eliminate artefacts, followed by full-wave rectification. The signal was then smoothed using a fourth-order 20 Hz low-pass filter. Subsequently, data were normalized based on each subject’s maximum electromyographic value, and the timing axis of the forehand smash was interpolated to 100 data points to eliminate the impact of variations in cycle duration.

#### 2.6.2 Muscle synergy extraction

The NMF algorithm is employed to extract muscle synergy features from electromyographic data (Cheung and Tresch, 2005; Rabbi et al., 2020). The muscle activity matrix D(t) is decomposed into time-invariant synergy vectors Wi (muscle weights) and time-varying activation coefficients Ci(t), with the reconstruction formula given by:

$$D\left(t\right)=\overset{N_{\text{syn}}}{\underset{i=1}{\sum }}C_{i}\left(t\right)W_{i}\;\left(N_{\text{syn}}\text{ Indicates the number of synergies}\right)$$

To mitigate the sensitivity of NMF to initial conditions and ensure the stability of the extracted synergies, for each subject we performed 50 independent runs of the algorithm with random initializations. The solution with the highest variance accounted for (VAF) was selected for subsequent analysis.

To determine the optimal number of synergies, iterative extraction of 1–14 synergies was performed, with the criterion being the minimum number of synergies required to explain over 90% of the variance in electromyographic reconstruction (VAF).

The VAF calculation formula is:

$$\text{VAF}=1‐\frac{\text{SSE}}{\text{SST}}$$

Where SST denotes the total sum of squares and SSE denotes the sum of squared errors. The NMF iteration is initialized between 0 and the maximum electromyographic value, ceasing when VAF exceeds 90% (Acuña et al., 2022; Kubota et al., 2021). The solution with the highest VAF is selected for subsequent analysis.

#### 2.6.3 Muscle synergy clustering and matching

To enable robust comparison of muscle synergies across participants and intervention groups (SG, RBG, ESG), a two-stage clustering and matching procedure was implemented. First, the muscle weight vectors (Wi) extracted via NMF from all participants within a given intervention group were pooled. K-means clustering (Munoz-Martel et al., 2021; Cheung et al., 2020) (using squared Euclidean distance, 1,000 repetitions) was applied to this pooled set to identify the representative synergy patterns (templates) specific to that group. The optimal number of clusters (k) for each group was objectively determined using the Gap statistic, ensuring the derived templates captured the dominant modular strategies elicited by each intervention. This process resulted in distinct sets of templates for the SG, RBG, and ESG groups, reflecting their potentially differing synergy counts.

To compare functionally similar modules across groups, the synergy templates from the SG group were designated as the reference set. The templates from the RBG and ESG groups were matched to this reference based on the Pearson correlation coefficient (r ≥ 0.6) of their muscle weight vectors. Templates that did not meet this correlation threshold were classified as group-specific synergies and analyzed separately. This approach enabled comparative analysis of conserved modular strategies across interventions while accounting for differences in synergy number and composition.

#### 2.6.4 Calculation of intermuscular time-frequency coherence

Intermuscular time-frequency coherence (TFC) was computed via a Python script within the short-time Fourier transform (STFT) framework (Halliday et al., 1995). Following bandpass filtering (20–400 Hz) and full-wave rectification of electromyographic signals, a 10-millisecond sliding window was employed to extract signal envelopes. Signals were segmented using a Hamming window (200 samples, 75% overlap) to compute cross-spectra and self-spectra. Following two-dimensional convolution smoothing, TFC was calculated using the following formula:

$$C_{xy}\left(l,f\right)=\frac{{\left|{\hat{p}}_{xy}\left[l,f\right]⊗v\left[t\right]\right|}^{2}}{\left\{{\left|{\hat{p}}_{xx}^{\wedge }\left[l,f\right]\right|}^{2}⊗v\left[t\right]\right\}\left\{{\left|{\hat{p}}_{yy}\left[l,f\right]\right|}^{2}⊗\;v\left[t\right]\right\}}$$

Where ⊗ denotes the convolution operation. TFC is normalized to the 0–1 range, with values closer to one indicating stronger coherence. Calculate and compare the significant coherence areas for trunk–lower limb and trunk–upper limb muscle pairs across the α (8–15 Hz), β (15–30 Hz), and γ (30–50 Hz) frequency bands.

#### 2.6.5 Statistical analysis

Data statistical analysis was conducted using SPSS 26.0 software. Outliers were removed via box plots, and data normality was verified using the Shapiro-Wilk test. Normally distributed data underwent repeated measures analysis of variance, followed by Bonferroni-corrected paired t-tests for multiple comparisons. Non-normally distributed data were analyzed using Friedman’s test, followed by Holm-corrected Wilcoxon signed-rank tests for multiple comparisons, with effect size r reported. Results are presented as mean ± standard deviation, with significance set at p < 0.05.

## 3 Results

### 3.1 Number of muscle synergies

When VAF values exceeded 0.9, the number of synergistic patterns across the three groups is presented in Table 3. Analysis of variance revealed statistically significant differences in synergistic pattern counts between groups (p < 0.001). Post-hoc comparisons revealed that the RBG exhibited significantly more synergistic patterns than both the SG (p = 0.005) and the ESG (p < 0.001). No significant difference was observed between the SG and ESG (p = 0.132).

**TABLE 2:** Experimental intervention protocol.

Item	SG	RBG	ESG
Movement pattern	Barbell squat	Resistance band lateral step exercise	Squat combined with electrical stimulation
Movement specifications	Feet positioned 1.2–1.5 times shoulder width apart, toes turned out 10°–15°, squatting until thighs form a 90°–100° angle with the ground	Resistance band secured above knee joint, maintaining tension during lateral movement	Electrical stimulation triggered at 90° knee flexion, sustained until movement completion
Load intensity	70% of 1RM (Lin et al., 2025)	Approximately 18% of body weight	70% of 1RM (Lin et al., 2025)
Electrical stimulation parameters	—	—	75Hz, 400 μs, 90% tolerable intensity
Sets and repetitions	3 sets × 3 reps	3 sets × 10 m	3 sets × 3 reps
Rest period between sets	2 min	2 min	2 min
Test timing	6 min post-intervention (Wilson et al., 2013)	6 min post-intervention (Wilson et al., 2013)	6 min post-intervention (Wilson et al., 2013)

**TABLE 3:** Number of synergists (mean ± SD).

	Squat	Resistance band	Electrical stimulation
Number of synergists	3.33 ± 0.52	5.0 ± 0.63	2.33 ± 0.82

### 3.2 Muscle activation weights in muscle synergy

**FIGURE 5:**
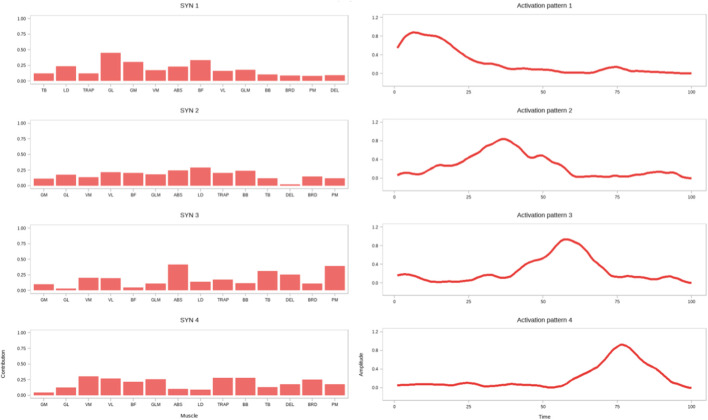
Muscle mass (left) and activation curves (right) for synergistic muscle groups in the SG.

**FIGURE 6:**
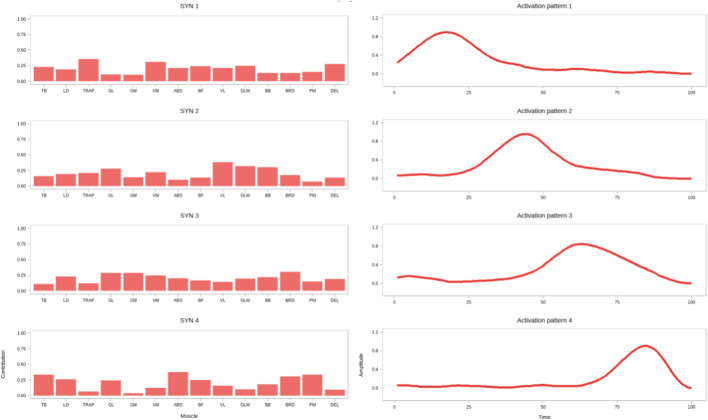
Muscle mass (left) and activation curves (right) for synergistic muscle groups in the RBG.

**FIGURE 7:**
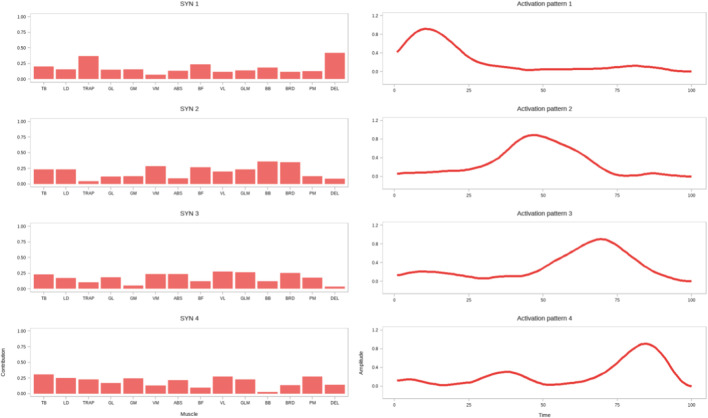
Muscle weight (left) and activation curves (right) for each synergistic muscle group in the ESG.

**FIGURE 8:**
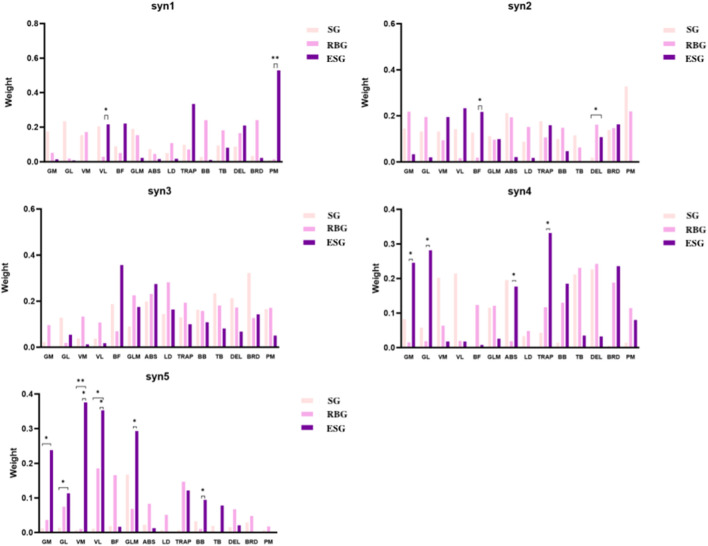
Comparison of muscle weights across three groups. * Indicates p ≤ 0.05; ** indicates p ≤ 0.01.

Regarding muscle activation weights (Figures 5–7), in SYN1, the trapezius (TRAP) and pectoralis major (PM) were the primary activated muscles (Figure 8; Table 4). Comparing the RBG with the ESG, the RBG exhibited significantly higher activation weights for the vastus lateralis (VL) and pectoralis major (PM) muscles (p < 0.05).

**TABLE 4:** Weighting for SYN muscle activation.

Muscle	SYN	Squats	Resistance bands	Electrical stimulation	SYN	Squats	Resistance bands	Electrical stimulation
GM	SYN 1	0.18 ± 0.22	0.05 ± 0.10	0.01 ± 0.03	SYN 2	0.14 ± 0.32	0.22 ± 0.33	0.03 ± 0.05
GL		0.23 ± 0.32	0.02 ± 0.04	0.01 ± 0.02		0.13 ± 0.22	0.19 ± 0.28	0.02 ± 0.03
VM		0.22 ± 0.26	0.19 ± 0.22	0.00 ± 0.00		0.13 ± 0.22	0.09 ± 0.17	0.19 ± 0.28
VL		0.20 ± 0.30	0.22 ± 0.26^c^	0.01 ± 0.03		0.14 ± 0.19	0.02 ± 0.02	0.23 ± 0.29
BF		0.09 ± 0.16	0.05 ± 0.07	0.22 ± 0.36		0.13 ± 0.16	0.22 ± 0.23^c^	0.01 ± 0.02
GLM		0.19 ± 0.38	0.15 ± 0.12	0.02 ± 0.05		0.11 ± 0.19	0.10 ± 0.13	0.10 ± 0.23
ABS		0.07 ± 0.10	0.04 ± 0.05	0.02 ± 0.04		0.21 ± 0.17	0.19 ± 0.27	0.02 ± 0.03
LD		0.05 ± 0.12	0.11 ± 0.14	0.02 ± 0.04		0.09 ± 0.10	0.15 ± 0.21	0.02 ± 0.03
TRAP		0.10 ± 0.20	0.07 ± 0.06	0.33 ± 0.36		0.18 ± 0.34	0.11 ± 0.18	0.16 ± 0.25
BB		0.03 ± 0.04	0.24 ± 0.25	0.01 ± 0.03		0.10 ± 0.17	0.15 ± 0.13	0.05 ± 0.12
TB		0.09 ± 0.18	0.18 ± 0.20	0.08 ± 0.18		0.12 ± 0.22	0.06 ± 0.14	0.00 ± 0.00
DEL		0.09 ± 0.10	0.17 ± 0.25	0.21 ± 0.22		0.11 ± 0.14^b^	0.16 ± 0.27	0.00 ± 0.00
BRD		0.03 ± 0.03	0.24 ± 0.26	0.02 ± 0.03		0.14 ± 0.19	0.15 ± 0.21	0.16 ± 0.35
PM		0.01 ± 0.02	0.53 ± 0.37^c^	0.00 ± 0.00		0.33 ± 0.31	0.22 ± 0.37	0.00 ± 0.00
GM	SYN 3	0.02 ± 0.03	0.10 ± 0.23	0.00 ± 0.00	SYN 4	0.08 ± 0.20	0.25 ± 0.31^c^	0.00 ± 0.00
GL		0.13 ± 0.31	0.02 ± 0.03	0.05 ± 0.08		0.06 ± 0.09	0.28 ± 0.28^c^	0.00 ± 0.00
VM		0.04 ± 0.05	0.13 ± 0.23	0.01 ± 0.02		0.20 ± 0.31	0.06 ± 0.10	0.02 ± 0.04
VL		0.04 ± 0.08	0.11 ± 0.09	0.02 ± 0.02		0.21 ± 0.32	0.02 ± 0.04	0.02 ± 0.04
BF		0.19 ± 0.20	0.07 ± 0.07	0.36 ± 0.40		0.00 ± 0.00	0.12 ± 0.15	0.01 ± 0.02
GLM		0.09 ± 0.12	0.22 ± 0.19	0.17 ± 0.27		0.11 ± 0.18	0.12 ± 0.13	0.03 ± 0.07
ABS		0.20 ± 0.17	0.23 ± 0.19	0.27 ± 0.38		0.20 ± 0.21	0.18 ± 0.19^c^	0.00 ± 0.00
LD		0.13 ± 0.25	0.19 ± 0.23	0.10 ± 0.10		0.04 ± 0.05	0.33 ± 0.31	0.00 ± 0.00
TRAP		0.40 ± 0.16a	0.04 ± 0.06	0.29 ± 0.22		0.17 ± 0.19	0.25 ± 0.21^c^	0.12 ± 0.13
BB		0.16 ± 0.10	0.16 ± 0.32	0.11 ± 0.09		0.01 ± 0.02	0.13 ± 0.12	0.18 ± 0.29
TB		0.23 ± 0.26	0.18 ± 0.20	0.08 ± 0.18		0.21 ± 0.28	0.23 ± 0.32	0.04 ± 0.09
DEL		0.21 ± 0.30	0.17 ± 0.20	0.07 ± 0.17		0.23 ± 0.27	0.24 ± 0.22	0.03 ± 0.08
BRD		0.32 ± 0.29	0.13 ± 0.12	0.14 ± 0.17		0.01 ± 0.01	0.19 ± 0.18	0.24 ± 0.36
PM		0.17 ± 0.30	0.17 ± 0.17	0.05 ± 0.10		0.01 ± 0.03	0.11 ± 0.10	0.08 ± 0.15
GM	SYN 5	0.00 ± 0.01	0.24 ± 0.20^c^	0.03 ± 0.07				
GL		0.01 ± 0.02^a^	0.11 ± 0.08	0.07 ± 0.17				
VM		0.00 ± 0.00^a^	0.38 ± 0.31^c^	0.00 ± 0.00				
VL		0.00 ± 0.01	0.35 ± 0.28	0.00 ± 0.01				
BF		0.01 ± 0.03	0.17 ± 0.35	0.02 ± 0.04				
GLM		0.16 ± 0.39	0.29 ± 0.24^c^	0.01 ± 0.03				
ABS		0.02 ± 0.04	0.08 ± 0.12	0.01 ± 0.03				
LD		0.00 ± 0.00	0.05 ± 0.11	0.00 ± 0.00				
TRAP		0.00 ± 0.00	0.15 ± 0.14	0.12 ± 0.30				
BB		0.03 ± 0.06	0.09 ± 0.08^c^	0.00 ± 0.00				
TB		0.01 ± 0.03	0.00 ± 0.00	0.08 ± 0.19				
DEL		0.01 ± 0.02	0.07 ± 0.11	0.02 ± 0.05				
BRD		0.02 ± 0.05	0.05 ± 0.04	0.00 ± 0.00				
PM		0.00 ± 0.00	0.02 ± 0.03	0.00 ± 0.00				

^a^ Indicates a significant difference between the SG, and the RBG.

^b^ Indicates a significant difference between the SG, and the ESG.

^c^ Indicates a significant difference between the RBG, and the ESG.

In SYN2, the primary activated muscles were the pectoralis major (PM), rectus abdominis (ABS), and vastus medialis (VM). No significant differences were observed between the squat and RBGs; however, the deltoid (DEL) activation weight was significantly higher in the ESG compared to the SG (p < 0.05). When comparing the resistance band and ESGs, the biceps femoris (BF) activation weight was significantly higher in the ESG (p < 0.05).

In SYN3, the biceps femoris (BF) and brachioradialis (BRD) were the primary activated muscles. However, no significant differences in activation weights were observed across all muscles between the three groups.

In SYN4, the primary activated muscles were the gastrocnemius medial head (GL) and trapezius (TRAP). No significant differences were observed between the SG and the RBG. Comparing the RBG with the ESG, the latter exhibited significantly higher activation weights for the gastrocnemius medial head (GM), gastrocnemius lateral head (GL), rectus abdominis (ABS), and trapezius (TRAP) muscles (p < 0.05).

In SYN5, the primary activated muscles were the vastus medialis (VM), vastus lateralis (VL), and gluteus maximus (GLM). Compared to the SG, the ESG exhibited significantly higher activation weights for the gastrocnemius medial head (GM), gastrocnemius lateral head (GL), and vastus medialis (VM) (p < 0.05). Comparing the RBG with the ESG, the latter exhibited significantly higher activation weights for the vastus medialis (VM), vastus lateralis (VL), gluteus maximus (GLM), and biceps brachii (BB) muscles (p < 0.05).

### 3.3 Intermuscular coherence

**FIGURE 9:**
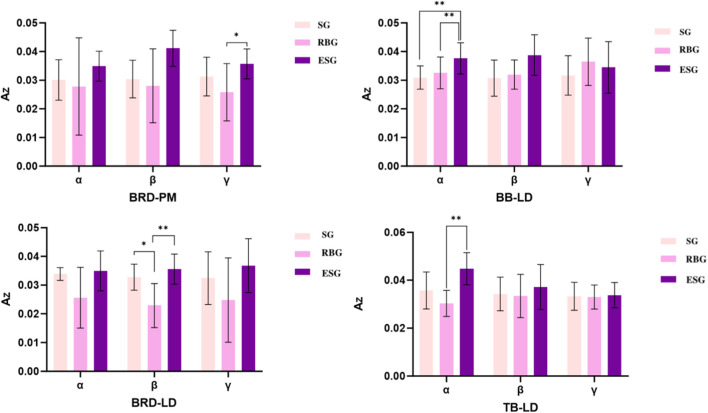
Comparative analysis of muscle group coordination between upper limbs and trunk.

**FIGURE 10:**
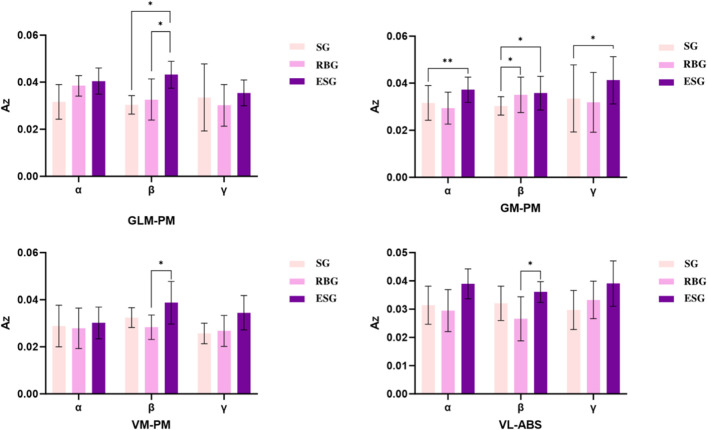
Comparative analysis of muscle group coordination between lower limbs and trunk.

In the intermuscular time-frequency coherence analysis (Supplementary Appendix1), comparisons between upper limb and trunk muscles revealed (Figures 9, 10):

- Within the γ band, the AZ value for the brachioradialis (BRD)-pectoralis major (PM) pair was significantly higher in the ESG than in the elastic band group (p = 0.02). Within the α band, the AZ value for the biceps brachii (BB)-latissimus dorsi (LD) pair was significantly higher in the ESG than in both the elastic band group (p = 0.007) and the SG (p = 0.002); the AZ value for the triceps brachii (TB)-latissimus dorsi (LD) pair was significantly higher in the ESG than in the elastic band group (p = 0.006). Furthermore, within the β band, the AZ value for the BRD-LD pair was significantly higher in the ESG than in the RBG (p = 0.006), and significantly higher in the SG than in the RBG (p = 0.03). Regarding comparisons between lower limb and trunk muscles, the AZ value for the GM-PM pair in the α band was significantly higher in the ESG than in the SG (p = 0.002). The AZ value for the GLM-PM pair in the β band was significantly higher in the ESG than in both the elastic band group (p = 0.04) and the SG (p = 0.01); Within the GM-PM pair, both the electrical stimulation and RBGs were significantly higher than the SG (p = 0.01). The AZ value for the VM-PM pair was significantly higher in the ESG than in the RBG; similarly, the VL-ABS pair showed a significant increase in the ESG compared to the RBG. Within the γ band, the AZ value for the GM-PM pair was significantly higher in the ESG than in the SG (p = 0.04).

## 4 Discussion

This study employed muscle synergy and intermuscular coherence analyses to investigate, from a neural control perspective, the effects of different post-activation potentiation (PAP) interventions on neuromuscular activation during the smash technique in badminton athletes recovering from meniscus injuries. Key findings are as follows:

The RBG exhibited significantly more muscle synergies than both the SG and ESG. This quantitative difference can be interpreted through complementary theoretical lenses. From an efficiency perspective, the increased synergy count may reflect a compensatory strategy where the nervous system employs a greater variety of modules to manage knee instability and the novel demands of elastic band training (Halliday et al., 1995). This pattern resembles the exploratory phase of motor learning, where the system tests diverse module combinations before consolidating optimal solutions (Safavynia et al., 2011). Concurrently, motor control theory offers an alternative interpretation: an increased number of synergies may represent a ‘freezing of degrees of freedom’ strategy (Clark et al., 2010). Under this framework, the nervous system could adopt a more constrained control architecture—activating more, but potentially more rigid, synergistic units—to prioritize joint stability and reduce movement variability during rehabilitation. Thus, the observed pattern in the RBG may signify an adaptive neuromuscular response focused on stability, rather than solely indicating an inefficient control state. In contrast, the ESG exhibited a synergy count comparable to the SG but demonstrated synergistic patterns characterized by higher efficiency in subsequent analyses. This suggests that neuromuscular electrical stimulation, through its precise neural drive, may effectively circumvent injury-induced inhibition (D’avella et al., 2003). It likely reinforces core synergistic pathways relevant to functional movements, thereby assisting the nervous system in more rapidly consolidating economical control modules and shifting focus from control ‘quantity’ to ‘quality’ (Doucet et al., 2012).

This study found that the ESG exhibited significantly higher activation weights for key stabilising and prime mover muscles of the lower limbs (such as GM, GL, VM, VL, GLM) across multiple synergistic modules, particularly SYN4 and SYN5. This finding holds particular significance within the context of recovery from meniscal injury. Common phenomena following meniscal injury, such as quadriceps inhibition, directly result in disruption of the lower limb kinetic chain and abnormal biomechanical patterns. SYN4 and SYN5 are typically closely associated with propulsion, stability, and force transmission during movement–precisely the areas where post-injury performance declines most markedly. This finding not only aligns with prior research but also profoundly illuminates the unique mechanism by which NMES counteracts injury-related neural inhibition. Chen et al. (2025) similarly observed that electrical stimulation training enhances lower limb muscle activation (Dos Anjos et al., 2023). Its profound value lies in NMES’s ability to effectively bypass spinal and supraspinal central inhibition triggered by injury and pain. By delivering external currents, it directly and synchronously activates numerous α-motor neurons, particularly high-threshold motor units rendered “dormant” by inhibition (Chen et al., 2025). This tonic contraction provides the central nervous system with robust and consistent proprioceptive feedback, which is crucial for remodelling movement commands disrupted by injury and re-establishing effective connections between brain and muscle (Karamian et al., 2022). Consequently, during subsequent voluntary smash actions, the CNS can more effectively drive these key muscle groups, manifested by a significant increase in their weighting within the synergistic module. This demonstrates the irreplaceable value of electrical stimulation in directly targeting the core of post-injury neuromuscular dysfunction, precisely reversing inhibition, and reconstructing normal force generation patterns.

Furthermore, the ESG exhibited significant advantages in alpha, beta, and gamma band coherence across multiple muscle pairs. For athletes with meniscal injuries, the damage not only affects local muscles but also disrupts multi-muscle coordination spanning joints and even limbs (Proske and Gandevia, 2012). Intermuscular coherence reflects shared neural drive sources, and its enhancement signifies improved neural coordination (Vanzile et al., 2022). This finding aligns strongly with prior research and points to NMES’s potential in facilitating the reorganization of higher-order neurological functions post-injury. Wand et al. (2025) noted that neuromuscular electrical stimulation can improve athletes’ neural coordination (Wang et al., 2025). This study further reveals that electrical stimulation not only enhances gamma-band coherence representing direct spinal cord conduction but also significantly elevates alpha and beta-band coherence levels, which are often associated with sensorimotor integration and postural control. These findings suggest that the PAP effect of electrical stimulation could support motor function, possibly by facilitating communication between cortical and subcortical structures involved in movement preparation and coordination (Carson and Buick, 2021). Intense peripheral electrical stimulation inputs provide the central nervous system, during its functional remodeling phase, with abundant high-quality and consistent proprioceptive signals. This may reinforce synchronized activity among distinct neuronal pools governing synergistic muscle groups (Simmons et al., 2021). For badminton players urgently requiring restoration of highly coordinated trunk-limb function during sudden stops, directional changes, and take-offs, this enhanced supraspinal coordination constitutes the neural foundation for safe, efficient return to competition and sustained elite performance.

Limitations: This study has several important methodological and population-related limitations. First, the use of a static maximum voluntary contraction (MVC) for EMG normalization, while standard for cross-condition comparisons, may not fully represent the dynamic neural drive characteristic of high-velocity ballistic movements such as the badminton smash. Second, the selection of muscle synergies was based on a global variance accounted for (VAF) threshold of ≥0.9, a conventional approach that facilitates cross-study comparison. However, this global criterion can be disproportionately influenced by high-amplitude muscles, potentially obscuring the reconstruction accuracy of individual muscles with lower activation levels and affecting the fine-grained interpretation of synergy structure. Third, while the loads for different intervention modalities were selected based on practical and evidence-based considerations, the presence of differing mechanical loads (e.g., between SG and ESG) introduces a confounding factor that complicates the isolation of the pure effect of neuromuscular electrical stimulation. Finally, the sample consisted exclusively of high-level male athletes recovering from meniscal injury, which may limit the generalizability of the findings to female athletes, non-athletic populations, or individuals with different knee pathologies. Future research should aim to employ dynamic normalization methods, incorporate more robust synergy extraction criteria (such as evaluating VAF per muscle or utilizing cross-validation), implement matched-load experimental designs, and recruit more diverse cohorts to further validate and extend these findings.

## 5 Conclusion

This study confirms that for high-level badminton athletes in the recovery phase of meniscal injury, neuromuscular electrical stimulation effectively overcomes joint-induced muscle inhibition through its precise neural drive characteristics, demonstrating unique advantages in neuromuscular system remodelling. Compared to the compensatory differentiation of control strategies induced by elastic band training and the limitations of conventional squat training, electrical stimulation intervention not only significantly enhances the contribution of key motor muscle groups within functional synergistic modules but also improves neuromuscular coordination across multiple frequency bands. This promotes functional reorganization of movement control patterns from the spinal cord level to subcortical and cortical levels. These findings indicate that neuromuscular electrical stimulation represents an effective rehabilitation strategy for achieving qualitative changes in neuromuscular control and optimising athletic performance.

## Data Availability

The original contributions presented in the study are included in the article/Supplementary Material, further inquiries can be directed to the corresponding author.
