# Supplementary material for: The effect of post-activation potentiation on neuromuscular activation of smashing technique during the recovery period of meniscal injuries in elite badminton players: non-negative matrix factorization-based muscle and time-frequency coherence
Source: Front Physiol. 2026 Mar 5;17:1752266. doi: 10.3389/fphys.2026.1752266 (PMC12999388; doi:10.3389/fphys.2026.1752266)
Supplement: Supplementary file 1 [file Supplementaryfile1.docx]

**Appendix1 Intermuscular time-frequency coherence analysis**

**Figure 1.** Correlation Analysis of SG


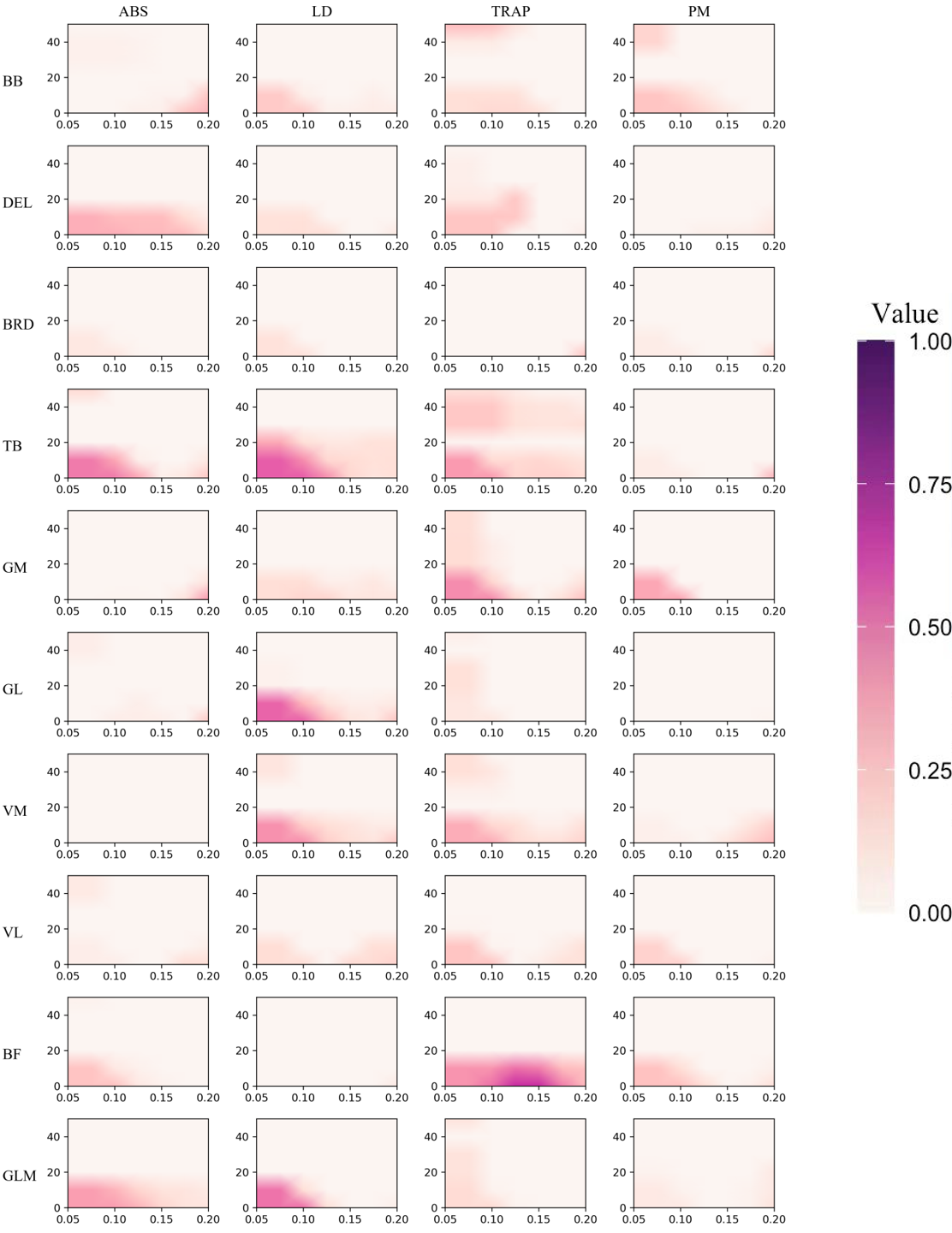


**Figure 2.** Correlation Analysis of RBG


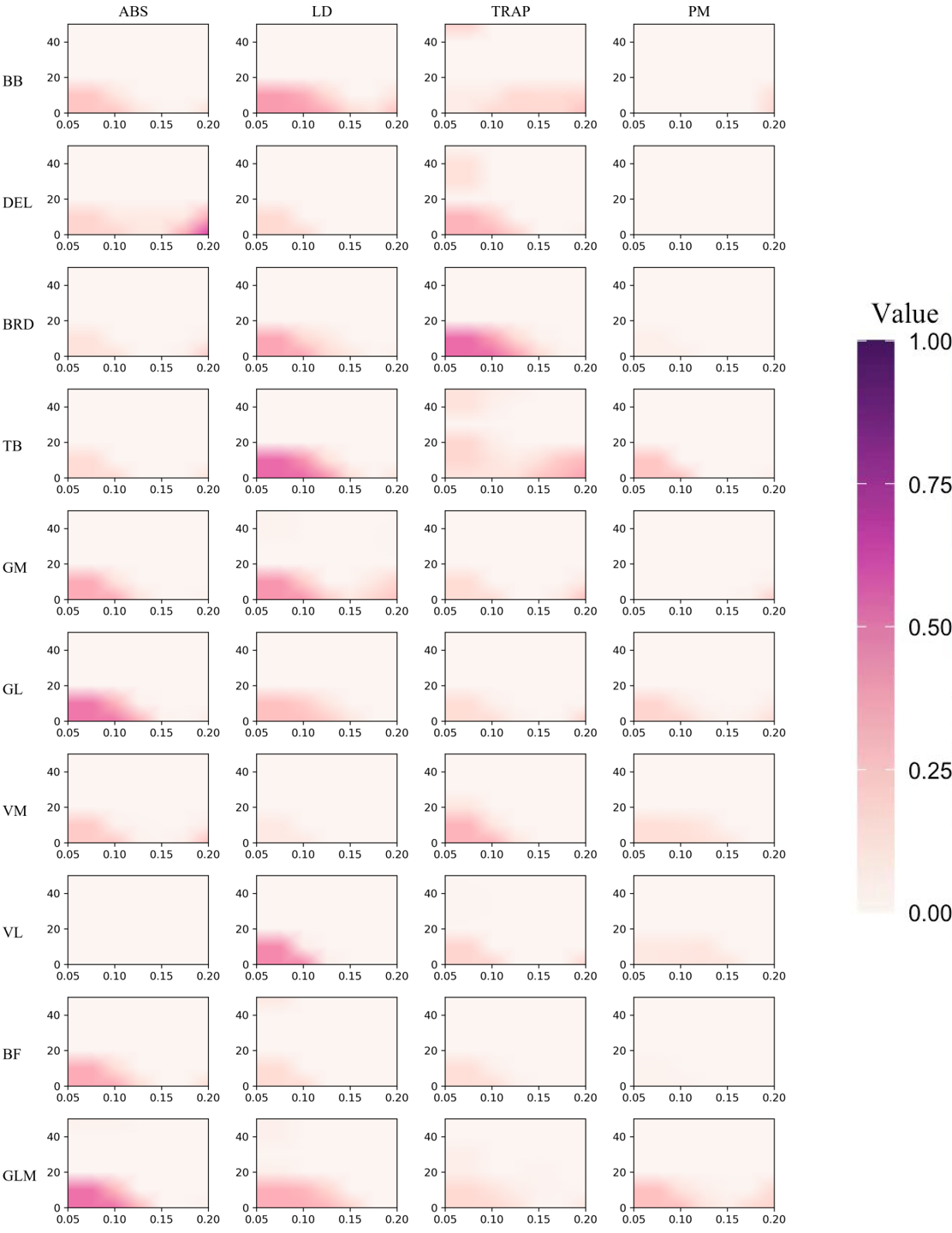


**Figure 3.** Correlation Analysis of ESG
